# CAMPAREE: a robust and configurable RNA expression simulator

**DOI:** 10.1186/s12864-021-07934-2

**Published:** 2021-09-25

**Authors:** Nicholas F. Lahens, Thomas G. Brooks, Dimitra Sarantopoulou, Soumyashant Nayak, Cris Lawrence, Antonijo Mrčela, Anand Srinivasan, Jonathan Schug, John B. Hogenesch, Yoseph Barash, Gregory R. Grant

**Affiliations:** 1 The Institute for Translational Medicine and Therapeutics, Perelman School of Medicine, University of Pennsylvania, Philadelphia, Pennsylvania USA; 2 Present address: National Institute on Aging, National Institutes of Health, Baltimore, Maryland USA; 3 Statistics and Mathematics Unit, Indian Statistical Institute, Bengaluru, Karnataka India; 4 Perelman School of Medicine, Enterprise Research Applications and High Performance Computing, Penn Medicine Academic Computing Services, University of Pennsylvania, Philadelphia, Pennsylvania USA; 5 The Institute for Diabetes, Obesity and Metabolism, The Department of Genetics, Perelman School of Medicine, University of Pennsylvania, Philadelphia, Pennsylvania USA; 6 Division of Human Genetics, Department of Pediatrics, Center for Chronobiology, Cincinnati Children’s Hospital Medical Center, Cincinnati, Ohio USA; 7 The Department of Genetics, Perelman School of Medicine, University of Pennsylvania, Philadelphia, Pennsylvania USA

**Keywords:** Simulation, Benchmarking, RNA-Seq

## Abstract

**Background:**

The accurate interpretation of RNA-Seq data presents a moving target as scientists continue to introduce new experimental techniques and analysis algorithms. Simulated datasets are an invaluable tool to accurately assess the performance of RNA-Seq analysis methods. However, existing RNA-Seq simulators focus on modeling the technical biases and artifacts of sequencing, rather than on simulating the original RNA samples. A first step in simulating RNA-Seq is to simulate RNA.

**Results:**

To fill this need, we developed the Configurable And Modular Program Allowing RNA Expression Emulation (CAMPAREE), a simulator using empirical data to simulate diploid RNA samples at the level of individual molecules. We demonstrated CAMPAREE’s use for generating idealized coverage plots from real data, and for adding the ability to generate allele-specific data to existing RNA-Seq simulators that do not natively support this feature.

**Conclusions:**

Separating input sample modeling from library preparation/sequencing offers added flexibility for both users and developers to mix-and-match different sample and sequencing simulators to suit their specific needs. Furthermore, the ability to maintain sample and sequencing simulators independently provides greater agility to incorporate new biological findings about transcriptomics and new developments in sequencing technologies. Additionally, by simulating at the level of individual molecules, CAMPAREE has the potential to model molecules transcribed from the same genes as a heterogeneous population of transcripts with different states of degradation and processing (splicing, editing, etc.). CAMPAREE was developed in Python, is open source, and freely available at https://github.com/itmat/CAMPAREE.

**Supplementary Information:**

The online version contains supplementary material available at 10.1186/s12864-021-07934-2.

## Background

High-throughput sequencing of RNA (RNA-Seq) has quickly become the standard method for assaying the population of transcripts in a given tissue or cell. As this technology continues to mature and evolve, many researchers have developed software tools to analyze sequencing data accurately and efficiently. Large-scale benchmarking studies to assess the relative performance of these programs across various stages in the RNA-Seq analysis pipeline help researchers navigate this fluid landscape of analysis tools [1–5]. While we would ideally perform benchmarking studies on real datasets where we know the true abundance of each transcript, it is not possible with current technologies to characterize the full transcriptome of any real sample with sufficient accuracy to achieve all critical goals of the benchmarking community. For this reason, simulated data provide an invaluable tool for the benchmarker’s arsenal.

Despite the strong demand for simulated data, there are only a few RNA-Seq simulators which generate data at the read-level. These include the FLUX simulator [6], BEERS [7], RSEM [8], and Polyester [9]. The FLUX simulator models some of the biases introduced during RNA-Seq library preparation but does not model polymorphisms such as indels and substitutions. BEERS simulates variants, errors, variable intron signal, and non-uniform sequence coverage, and despite being originally designed to benchmark aligners has since been adapted for use with transcript reconstruction and alternative splicing analyses [10, 11]. The simulator included with RSEM develops an empirical model of gene expression by quantifying real RNA-Seq datasets and using the expression parameters as the basis for simulation. Polyester, in addition to simulating coverage biases present in real data, is designed to generate full experiments with multiple biological replicates. It has also been adapted to include a model of GC-bias in coverage and for use with single-cell RNA-Seq data [12, 13]. While these simulators have been used in various benchmarking studies to great effect, they are primarily focused on capturing the biases introduced during RNA-Seq library preparation and sequencing. To date there has been little focus placed on realistic simulation of an RNA sample; the milieu of RNA molecules, often transcribed from a diploid genome, that serves as input for the molecular biology of library preparation and sequencing.

There are several advantages a molecule-level simulation of input RNA samples has over traditional generative models that are integrated directly into RNA-Seq simulators. First, it provides a more natural parallel to real RNA-Seq experiments, where purified RNA serves as input for the various molecular biology steps required to prepare and sequence the sample. Second, working at the level of individual molecules, rather than gene models, allows us to simulate the heterogeneous populations of RNA we expect to see in real samples. These include molecules produced from the same gene that are in different states of transcription, editing, splicing, and degradation [14–17]. Additionally, many RNA samples are collections of transcripts from heterogeneous sources like multiple cancer cell populations, unstable viral genomes, or contamination from microorganisms [18–21]. As we develop a greater appreciation for the clinical significance of these biological processes and develop new methods to search for them, it is critical that we also have simulated data that reflect these phenomena. Third, we gain greater fidelity in our ability to diagnose where and how analysis algorithms yield inaccurate results. If a particular set of reads fails to align, or incorrectly drives normalization and differential expression, we can trace the paths these reads took through the RNA-Seq protocol back to their precursor molecules and intermediates to look for common features. On the whole, molecule-level simulations will give us much tighter control over the parameters we use to develop and test analysis software and are likely to yield more realistic data.

Here we present the Configurable And Modular Program Allowing RNA Expression Emulation (CAMPAREE), a simulator designed to generate realistic RNA samples at the molecular level from diploid genomes. CAMPAREE works by calculating empirical distributions for gene, isoform, intronic, and allelic expression from real data, and producing a collection of full-length transcripts. These distributions are based on a given transcript annotation that must have both gene- and isoform-level information. The output of CAMPAREE is intended primarily to prime other RNA-Seq simulators, which will then model the biases introduced by library preparation and sequencing. Additionally, the model underlying this simulation makes CAMPAREE output useful for creating idealized representations of coverage plots, separating out signals across different isoforms and alleles. CAMPAREE is an open source program written in Python3 and freely available at https://github.com/itmat/CAMPAREE. Since CAMPAREE bases its gene expression on given annotations, it can simulate any form of RNA (short, long, etc.) that is reflected in the input annotation.

## Implementation

### CAMPAREE configuration and input

The CAMPAREE pipeline uses a configuration file to give users detailed control over the inputs, outputs, and execution of the simulation. This includes two important options: the ability to specify the job scheduler if running in a cluster environment, and a seed value for random number generation. CAMPAREE currently supports Load Sharing Facility (LSF) and Sun Grid Engine (SGE) cluster environments, as well as serial execution on a single machine. The seed value is critical for reproducibility, as two CAMPAREE runs initiated with the same seed value and input files will yield identical results (even if they are run serially or using a different job scheduler). If a user does not specify a seed value when running the pipeline, CAMPAREE will report a seed value that can be used to duplicate its results. We recommend that users include these seed values when reporting any CAMPAREE results.

In order to run successfully, CAMPAREE requires an organism’s genome sequence in FASTA format, a gene/transcript annotation in GTF format, and a file indicating the ploidy of chromosomes for each gender of the organism (if the user wants to generate diploid data). Using these files, CAMPAREE will generate a genome index with STAR v2.5.2a [22], although the user can provide a pre-built index as long as it is compatible with the same version of STAR. For individual simulation runs, CAMPAREE accepts a pair of files per simulated sample containing raw, unnormalized and unaligned paired-end RNA-Seq reads in FASTQ or FASTA format. Typically, these will consist of a related set of samples (e.g. two sets of samples from different experimental conditions). The current version of CAMPAREE requires raw input data (FASTQ, and optional BAM files) from paired-end, strand-specific sequencing experiments. Also, in order to simulate diploid data, CAMPAREE currently requires that each input sample is generated from a single, individual organism rather than a pool. CAMPAREE begins by using STAR to align these FASTQ files to the reference genome index. Users may also provide their own genome-aligned BAM files to skip this step, as long as the alignments in the BAM file are sorted in chromosome coordinate order. See Additional file 1 for a table listing the inputs and outputs for each step in the CAMPAREE pipeline.

### Variant finding and phasing

In order to simulate diploid genomes for each of the input samples, CAMPAREE identifies genetic variants represented in the input data. The variant finder step takes the indexed BAM file generated by the genome alignment step and parses all the alignments to identify loci that differ from the reference by single-nucleotide variants (SNVs), insertions, or deletions. For variant calling, reads are discarded if they map to multiple locations in the reference. Additionally, if multiple reads have identical alignments, they are treated like PCR duplicates and collapsed down to a single alignment for counting purposes. Insertions and deletions in a given alignment are identified from ‘I’ and ‘D’ entries in the CIGAR string, respectively. SNVs are identified as single bases that differ from the reference base. For a variant to be retained, it must be represented in at least 3 % of the distinct, uniquely-aligned reads overlapping the locus. Lastly, no variant is retained if it is present in only a single alignment. Once CAMPAREE compiles the list of variants for each sample, it uses BEAGLE v5.0 [23] to perform haplotype phasing (i.e. determine which variants are on the same parental chromosome together). Note that BEAGLE requires at least two samples to perform this phasing operation, so users that wish to simulate data from diploid genomes must provide at least two input samples, or their own phased VCF file.

### Generating parental genomes and transcript annotations

Through the combination of variant finding and phasing, CAMPAREE generates two lists of variants for each sample that correspond to the two parental copies of each autosome. For each sample, the pipeline applies these variants to the reference sequence to create two full parental genome sequences. Insertions and deletions that are introduced into the parental genomes will change the number of nucleotides and shift the chromosomal coordinate systems relative to the reference genome. To account for these changes, CAMPAREE adjusts the coordinates in the input gene annotations to account for insertions and deletions, which creates separate reference annotations for each parent.

### Estimating intron, gene, transcript PSI, and allelic distributions

To ultimately determine the composition of simulated molecules in the final output, CAMPAREE estimates several empirical distributions from each sample. The pipeline determines the intronic distribution by first identifying all regions that are exclusively intronic (mintrons; intronic regions with no overlapping exonic regions from other genes or isoforms from the same strand). Next, for each mintron, CAMPAREE counts the number of uniquely-aligned reads/fragments from the genome-aligned data that overlap each mintron and normalizes these counts by mintron length in kilobases. This procedure is repeated for the antisense signal in each mintron, as well as the intergenic regions between genes. See Additional file 2 for a flowchart depicting this procedure. For the gene abundance and transcript percent splicing included (PSI) distributions (Additional file 3), CAMPAREE uses kallisto v0.45.0 [24] to map the raw input reads to the first parental transcriptome. Since allele-specific quantification is handled in a separate step and the two parental genomes are relatively similar, the pseudo-alignment results from the first parental transcriptome are sufficient to determine the transcript distribution. The kallisto results yield transcript-level estimated read counts (‘est_counts’ column in kallisto output), which CAMPAREE normalizes by the effective length of each transcript (‘eff_length’ column in kallisto output). Next, CAMPAREE uses these length-normalized transcript-level read counts to generate the gene-level distribution by summing the read counts across all transcripts associated with a given gene. This is a common approach for getting gene-level data from pseudo-aligners, which previous work has shown is more accurate than calculating gene-level read counts directly from genome-aligned reads [25]. For the transcript PSI distribution, CAMPAREE again identifies which transcripts are associated with a given gene and then divides each transcript-level read count by the gene-level read counts. The PSI values for the transcripts associated with a given gene will sum to one and represent the relative abundances of each isoform. Lastly, to generate the gene-level allelic distribution (Additional file 4), CAMPAREE uses Bowtie2 v2.3.4.3 [26] to align the raw input reads to each of the parental transcriptomes. For each read in the input, the pipeline compares the alignments to both parental transcriptomes and increments a gene-level read count for each parent depending upon the alignment results. If the read aligns to only one of the parental transcriptomes, the read count for the corresponding parental allele is incremented by one. If the read aligns to both parental transcriptomes, the read count for the parental allele with the smallest alignment edit distance is incremented. If the read aligns equally well to both parental transcriptomes, the read counts for both parental alleles are incremented by 0.5. After parsing the read alignments, CAMPAREE calculates the allelic imbalance for each gene and parental allele as the fraction of read counts for that allele over the total read count for both parental alleles. These two fractions for a given gene will sum to one and represent the relative abundances of transcripts originating from either parental allele. Once again, users may provide their own distributions instead of estimating them from the input data.

### Generating simulated molecules

For the final stage in the pipeline, CAMPAREE uses these empirical distributions together in a hierarchy to generate the molecules in the simulated sample. The pipeline repeats the following process until it generates the desired number of simulated molecules: 1) Randomly select a gene from the gene distribution. 2) Randomly select a parental allele from the allelic distribution for this gene. 3) Randomly select an isoform for this gene from the transcript PSI distribution. 4) Randomly determine whether to generate an unspliced version of the transcript (i.e. pre-mRNA containing all introns) by calculating the likelihood of pre-mRNA as the ratio of intronic read counts per base (from the intron distribution) to the gene-level read counts per base. By using average read counts per base, this accounts for differences in length between a gene’s introns and exons. 5) Having selected which transcript to generate, retrieve the full nucleotide sequence for the transcript and generate CIGAR strings mapping the transcripts back to the appropriate parental genome and the original reference genome. 6) Add a polyA tail to the 3ʹ end of the sequence and corresponding entries for soft-clipped bases (denoted by ‘S’) to the appropriate ends of each CIGAR string. Lastly, CAMPAREE saves this list of simulated molecules in either FASTA format, listing the full sequence and identifier for each molecule, or in a tab-delimited “molecule file” that lists the molecule sequence along with other metadata, including the reference and parental CIGAR strings.

### Datasets used in this manuscript and packaged with CAMPAREE

All CAMPAREE data presented in this manuscript were primed with two real mouse liver RNA-Seq samples, 9576 and 9577, from a previous study [27]. The FASTQ files for these samples are available from the Gene Expression Omnibus (9576 – GSM2599715; 9577 – GSM2599721). CAMPAREE was run with default parameters (seed value = 100) in an LSF environment using the genome sequence from the Ensembl GRCm38 build of the mouse genome and gene/transcript models from the Ensembl release 102 annotation [28].

For testing and diagnostic purposes, CAMPAREE is packaged with a reduced genome sequence and annotation collectively called the “baby genome.” The baby genome sequence is a subset of the Ensembl GRCm38 build of the mouse genome covering the following chromosome spans: chr1:57,943,156–58,943,156, chr2:26,644,480–27,644,480, chr3:88,196,078–89,196,078, chrM:1-16299, chrX:73,654,661–74,654,661, and chrY:663,558–1,744,052. Similarly, the baby genome annotation is a subset of gene/transcript models from the Ensemble release 93 [29] annotation that come from the same coordinate spans as the baby genome sequence. CAMPAREE also includes two test samples that are subsets of the full 9576 and 9577 samples described above. Reads were sampled from the original FASTQ files such that ~ 82 % of the read will map to the baby genome and the remaining ~ 18 % will not.

### Generating simulated data with BEERS, Polyester, and RSEM from CAMPAREE output

CAMPAREE was run on the full 9576 and 9577 samples as described above. The molecule file generated from sample 9576 was prepared for use by BEERS [7], Polyester [9], and RSEM [8] by the molecule_file_to_fasta_and_count_table.py script, packaged with CAMPAREE. This script accepts a molecule file as input and generates a FASTA file of unique transcript sequences contained in the molecule file, and a count matrix listing the number of occurrences for each sequence. This script was run with the ‘-s’ and ‘-t’ command line parameters to generate separate output files for each parental genome and to trim polyA tails from the transcript sequences and CIGAR strings, respectively.

To simulate reads with BEERS, separate sets of config files were generated from each of the parental genomes and annotations created by CAMPAREE. Briefly, the geneinfo, genenseq, and intronseq config files were created from the parental genome FASTA and the annotation using the standard set of scripts included with BEERS. The featurequantification config file was created from the matrix of sequence counts using the standard scripts included with BEERS. Next, BEERS was run separately for each parental genome using the two sets of config files. These reads were generated without any indels, errors, or intron retention, using the following command-line parameters: -strandspecific -error 0 -subfreq 0 -indelfreq 0 -intronfreq 0 -palt 0.

To simulate reads with Polyester v1.26.0, the simulate_experiment_countmat(seed = 100, strand_specific = TRUE) command was run in R v4.0.3. This command was given the FASTA file of transcript sequences and the matrix of counts, generating simulated reads from both parental alleles in a single run. Polyester was run using its default sequencing error model, so its simulated reads contain errors and indels, while the BEERS reads do not.

To simulate reads with RSEM v1.3.3, the FASTA file of transcript sequences was used to create an RSEM transcriptome index, where each transcript from each allele was treated as a separate gene. The rsem-prepare-reference command was run with the --bowtie2 and --bowtie2-path options, the latter of which pointed to the bowtie2 binary packaged with CAMPAREE. In order to simulate reads, RSEM requires a ‘sample_name.model’ file which defines empirical models for RNA-Seq read length, fragment length, sequencing errors, and quality scores. To generate this model file, the rsem-calculate-expression command was used to quantify the input FASTQ files (sample 9576) with the RSEM transcriptome index prepared above. The command was run with the following options: --paired-end --strandedness reverse --bowtie2 --bowtie2-path, the last of which pointed to the bowtie2 binary packaged with CAMPAREE. The count matrix and parental genome annotations produced by CAMPAREE were used to prepare an rsem genes.results file, where the expression of transcripts from each allele was represented in units of TPM (Transcripts per million). Lastly, simulated RNA-Seq reads were generated by running the rsem-simulate-reads command with the transcriptome annotation, model file, and genes.results file prepared above. The simulation command was run with the theta parameter set to 0, and with the ‘--seed 42’ parameter, for reproducibility. These simulated reads also contained sequencing errors and indels, as RSEM used a model file prepared from real data.

The FASTA/FASTQ files of simulated reads produced by all simulators were aligned to the Ensembl GRCm38 build of the mouse genome using STAR v2.5.2a. Coverage plots (in bedgraph format) were built from the resulting SAM files using the sam2cov tool v0.0.5.4-beta, available at https://github.com/khayer/sam2cov. STAR was run with the “--outSAMunmapped Within KeepPairs”, which is required by the sam2cov tool. Coverage plots for CAMPAREE output were generated by CAMPAREE itself, using the molecules_to_cov.py script in the BEERS_UTILS package (one of CAMPAREE’s requirements). Bedgraph files were visualized in the UCSC genome browser [30] and the STAR alignments were visualized directly in the IGV v2.5.2 [31].

## Results and discussion

### The CAMPAREE workflow

**Fig. 1:**
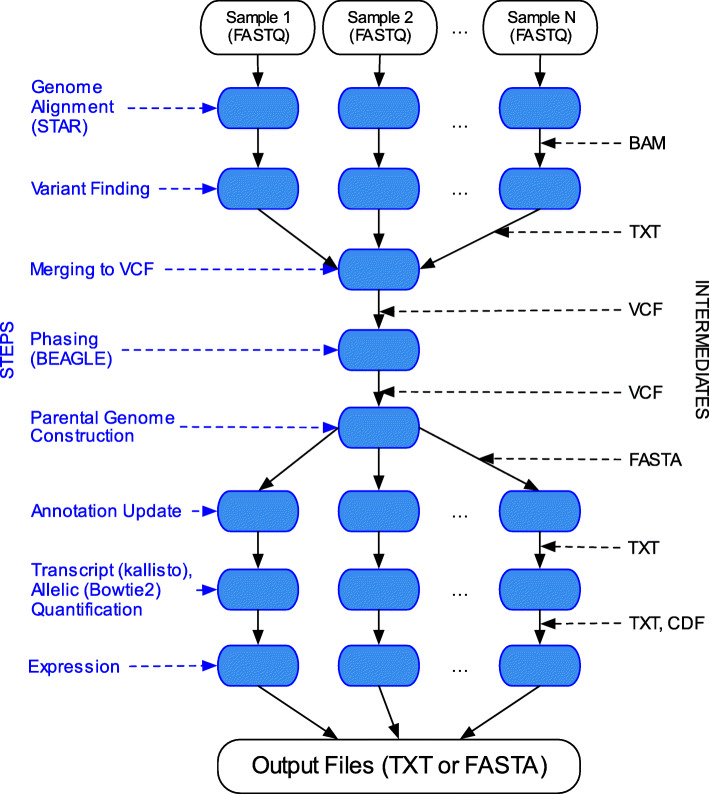
Flow chart of the CAMPAREE pipeline. Diagram of flow through the CAMPAREE pipeline from FASTQ file input (top) to molecule file or FASTA output (bottom). Names for each step listed on the left side. File types for intermediates between each of the steps listed on the right side

The CAMPAREE pipeline (Fig. 1) is based on estimating genetic variants and empirical distributions for gene, transcript, intron, and allele-specific expression from real RNA-Seq data (see Implementation section for full details). For this reason, CAMPAREE accepts raw FASTQ files from real RNA-Seq samples as input. Briefly, CAMPAREE begins by using STAR [22] to align raw reads from each sample to the appropriate reference genome, and then identifying the variants contained in those alignments that differ from the reference. These genome alignments also provide estimates for intron-level expression. Note that while CAMPAREE is configured by default to estimate variants and distributions directly from the raw FASTQ data, users may fully specify these distributions themselves, using their preferred tools for variant calling or some distributions of interest. After filtering the variants for likely sequencing errors, CAMPAREE combines the variants across samples and uses BEAGLE [23] to infer haplotype phasing. The pipeline uses the phasing information, along with the given reference sequence and annotation, to generate two parental genomes and transcriptomes for each sample. Next, for each input sample the pipeline realigns the raw FASTQ reads to both parental transcriptomes using kallisto [24] and Bowtie2 [26]. The kallisto data are used to estimate both the gene-level expression distribution, as well as the relative abundance of each isoform for a given gene. While kallisto can rapidly assign reads to their appropriate transcripts, it is a pseudo-aligner and therefore does not return complete, nucleotide-level alignments. Since most differences between the two parental transcriptomes are sequence-level features like single-nucleotide variants (SNVs) and indels, CAMPAREE uses the Bowtie2 alignments to assess which of the two parental alleles each read is most likely to originate from. Lastly, the pipeline simulates full-length RNA molecules according to the empirically-derived distributions (see Additional file 5 for the distributions estimated from two real samples). Given the total number of molecules to generate, as specified by the user, CAMPAREE uses the gene- and transcript-level distributions to determine the relative ratios of each RNA molecule, the allelic distribution to determine which parental genome each molecule originates from, and the intron distribution to determine what proportion of unspliced RNA molecules to generate. Each molecule generated by CAMPAREE lists its full sequence (including polyA tail, if added), transcript ID, parent of origin, chromosome, reference genome start coordinate, and parental genome start coordinate, as well as CIGAR strings mapping the molecule back to both the parental genome and the original reference genome. These two CIGAR strings are intended to facilitate the rapid mapping of molecules back to their source locations (common practice when assessing read/sequence alignment). CAMPAREE is capable of outputting each simulated RNA sample in a ‘molecule_file’ format, intended for use with future molecule-based RNA-Seq simulators, or in FASTA format, allowing for use with current RNA-Seq toolsets and simulators.

### Idealized coverage

**Fig. 2:**
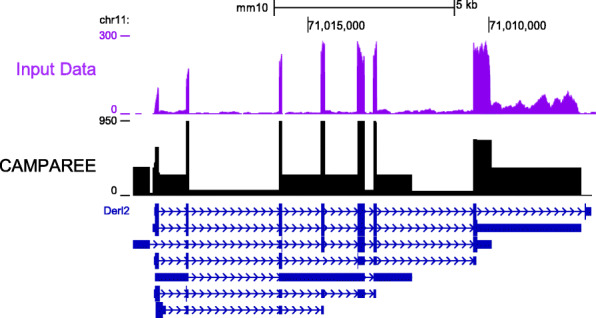
Idealized coverage plots from CAMPAREE output. Representative coverage plots of real, STAR-aligned input data (pink, top) and CAMPAREE (idealized) output primed from the input data (black, bottom). Transcript models for the gene *Derl2* displayed below coverage plots in dark blue. Image captured from the UCSC genome browser. Input and CAMPAREE data were generated from a mouse liver sample (9576; GSM2599715)

RNA-Seq coverage plots are one of the most ubiquitous methods for visualizing the results of an RNA-Seq experiment. These coverage plots, often viewed with the UCSC genome browser [30] or IGV [31], display characteristic non-uniform patterns of peaks and valleys over the length of a gene [12, 32]. Furthermore, the expressed regions displayed in a coverage plot only represent the ends of the underlying RNA fragments. Since typical RNA-Seq experiments are only reading the terminal 100–150 bp of fragments that are often more than twice this length, coverage plots for a paired-end experiment often have dead spaces in coverage between the forward and reverse reads. Technical artifacts, like these, combine to obscure the true expression patterns, making it difficult to discern which transcript regions are expressed. This is particularly true for loci that contain multiple, overlapping transcripts because the coverage represents a combination of the individual isoforms/alleles. CAMPAREE provides a solution to this problem. Since CAMPAREE estimates its abundance parameters from real data, the simulated molecules it generates have the side effect of providing an idealized view of RNA-Seq coverage. In other words, a user starts with an RNA-Seq FASTQ file and ends up with a clean representation of the signal meted out to individual isoforms and alleles. With this information, users can represent the data with idealized coverage plots that display only full-length isoforms and perfect signal, separated by isoform and allele (Fig. 2). To facilitate this feature and to provide another means of examining simulated data for benchmarking studies, CAMPAREE includes utilities to generate coverage plots from its output molecules.

### Enhancing existing RNA-Seq simulators

**Fig. 3:**
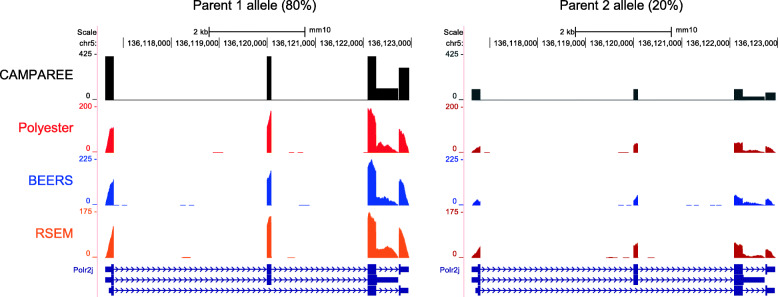
CAMPAREE adds allele-specific expression support to BEERS, Polyester, and RSEM. Representative coverage plots of simulated RNA-Seq data created by Polyester (red), BEERS (blue), and RSEM (orange) from CAMPAREE output (black). Separate coverage plots for signal from each parental allele are displayed on the left and right. Transcript models for the gene *Polr2j* displayed below coverage plots in dark blue. Note, Polyester, BEERS, and RSEM depth of coverage appears lower than CAMPAREE because they are displaying coverage from short reads, rather than full length transcripts. Image captured from the UCSC genome browser

**Fig. 4:**
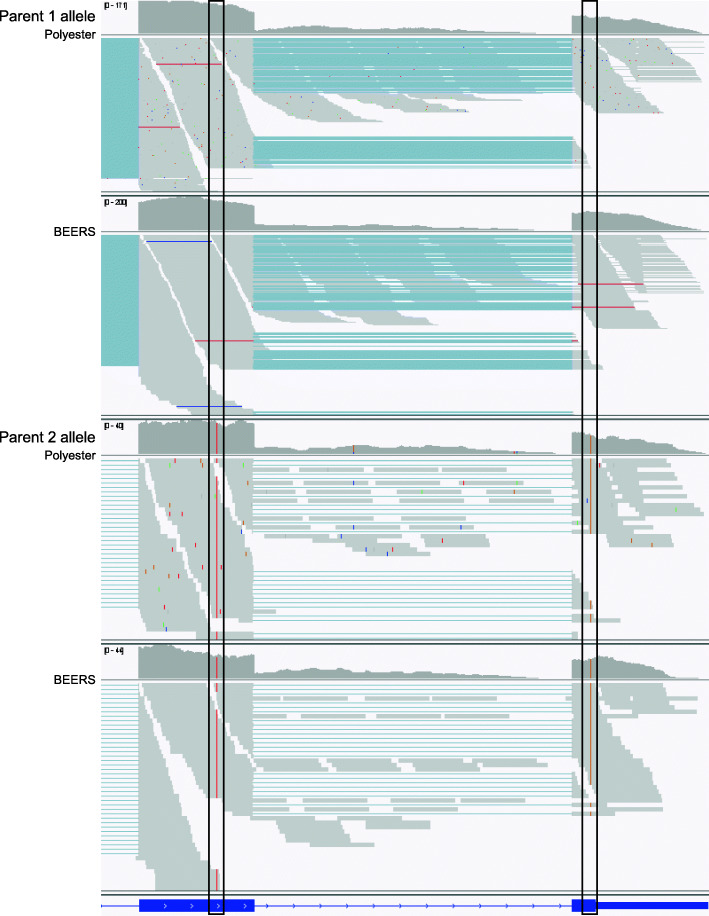
Variants introduced by CAMPAREE are maintained in BEERS and Polyester output. Coverage plots and alignments for reads simulated by BEERS and Polyester from the two terminal exons of *Polr2j*. Black rectangles highlight variants specific to each parental allele. Red lines on left indicate a ‘T’ substitution present in all alignments from parent 2 allele. Orange lines on the right indicate a ‘G’ substitution present in all alignments from parent 2 allele. Similar results for RSEM are displayed in Additional file 7

The goal behind the CAMPAREE simulator is to provide a realistic collection of simulated RNA molecules that represent the starting material for creating an RNA-Seq library. To demonstrate the utility of this approach, we used output from the CAMPAREE simulator to prime three existing simulators, BEERS [7], Polyester [9], and RSEM [8], to generate *diploid* simulated data. Neither BEERS nor Polyester simulate diploid data natively. While RSEM can be configured to simulate allele-specific RNA-Seq data, it requires an existing set of allele-specific transcript sequences as input and does not infer this information itself. To support RNA-Seq simulators which are not designed to take single-molecule input, CAMPAREE can output a FASTA file containing all the unique transcript sequences in a simulated sample, along with a count table listing the number of copies for each sequence. Polyester accepts both of these files directly as input for generating simulated RNA-Seq reads. For BEERS, we used the parental genomes and annotations generated by CAMPAREE, in combination with the transcript count table to prime the simulator. To prepare RSEM simulations, we used the parental annotations generated by CAMPAREE, along with the transcript count table and transcriptome FASTA. To simulate RNA-Seq data, RSEM also requires a model file which defines models for sequencing errors, quality scores, read lengths, and RNA fragment lengths estimated from real data. We generated this file by using RSEM to quantify the original FASTQ files used to run CAMPAREE, against the transcriptome containing sequences from both parental alleles. The transcript sequences output by CAMPAREE track the parental genome from which they originated. As a result, even though neither BEERS nor Polyester were originally designed to support allele-specific simulations from diploid genomes, priming them with CAMPAREE output confers this functionality (Fig. 3, Additional file 6). Looking broadly at read counts simulated by each tool (Additional file 6), both BEERS and RSEM show slight departures from the quantified transcript counts they were primed with, while Polyester almost exactly recapitulates the input quantities. This is reflective of the different approaches the simulators take to generating data. Polyester has an option allowing users to specify exact expression values for each transcript. Both BEERS and RSEM use the given quantification values to create probability distributions of transcript expression. They then sample from these distributions repeatedly when choosing which transcripts to simulate. This distinction between these simulators may be important depending upon the level of control users require for their specific needs. Comparing representative coverage plots of *Polr2j* from the CAMPAREE output to the BEERS, Polyester, and RSEM output, we see that all RNA-Seq simulators layered the coverage artifacts and edge effects we expect from real data on top of the CAMPAREE output. Additionally, we see representation of the same alternative splice forms across all simulators and a decrease in the overall depth of coverage for the second parental allele, which reflects the allele-specific expression pattern of this gene. Also, since CAMPAREE includes unspliced pre-mRNA transcripts in its output (indicated by the lines of coverage in the CAMPAREE plots that extend through the entire intronic regions), all three simulators include a low level of intronic expression. While BEERS can already introduce intronic expression into its gene models, this feature is not natively supported by Polyester or RSEM. If we zoom in on the terminal exons of *Polr2j* to alignment resolution, we can see the variants distinguishing the two parental alleles represented in reads generated by all three simulators (Polyester and BEERS - Fig. 4; RSEM - Additional file 7). Despite being developed as the basis for molecule-based RNA-Seq simulations, CAMPAREE is capable of introducing additional functionality and control to three existing RNA-Seq simulators.

## Conclusions

Arguably one of the most difficult aspects of simulating RNA-Seq data is generating a realistic RNA population to serve as the basis for the input sample. However, this problem has been largely overlooked by the community, where efforts tend to focus on simulating the biases introduced by library preparation and sequencing. To this end, we developed the CAMPAREE simulator to generate realistic RNA samples following empirical distributions derived from real RNA-Seq datasets. While this functionality may be useful to most users, we have also included the ability to explicitly specify the genetic variants and expression distributions (gene, transcript, intron, allelic) CAMPAREE uses to generate allele-specific data. CAMPAREE was developed in Python3, is open source, and freely available at https://github.com/itmat/CAMPAREE.

The gene-gene interaction networks and dependency structures underlying expression data present another challenge for simulations. These networks are highly complex, and can change drastically depending upon stimuli or cell states of the system of interest [33, 34]. Additionally, these interactions are often more complex than pairwise gene-gene interactions, involving input from many genes, which makes them difficult to both accurately detect and model at scale [35]. To make the analysis and simulation of expression data more tractable, many studies have modeled expression across multiple genes as an independent, identically distributed (iid) process [36, 37]. This is particularly problematic for benchmarking studies if the simulated data were generated using the same iid model and distributions as the analysis tools being tested. To avoid these potential confounds, CAMPAREE, as well as other recent benchmarking studies, including those using the RSEM simulator, have taken an empirical approach [13, 37]. By estimating gene expression values from real samples and then using these estimates as the ground truth in simulations, we can retain much of the complexity of real data, while also maintaining full knowledge of and control over the simulated molecules and transcript expression levels. While we hold the position that mimicking real data is an effective means to achieve more realistic simulated data, we also believe that effective benchmarking studies should involve both real and simulated data.

Most previous RNA-Seq simulators, including the ones developed by our group, focused on accurately modeling the molecular biology and biases of the library preparation and sequencing reactions. These simulators operate by using some underlying distribution (either statistically- or empirically-derived) to select a transcript to express. They then extract the full sequence for the transcript from a given annotation and apply some approximation of library preparation (i.e. fragmentation, size selection, PCR bias) to ultimately get the simulated read derived from that transcript. Here we focus on accurately modeling the input sample separately from library preparation, which we feel offers greater overall flexibility. By divorcing input sample simulation from library preparation/sequencing simulation, future simulators that operate on a given population of simulated molecules will be agnostic to genome sequence, annotations, and composition of the simulated input sample. For example, if researchers have access to multiple simulators, each modeling a different library prep technique (e.g. TruSeq, Smart-Seq2) or sequencing technology (e.g. Illumina, PacBio), they could use the same simulated sample as input across all programs. Furthermore, by priming different RNA-Seq simulators with the same input sample, we can compare their outputs to assess their performance and bias models. Perhaps most importantly, this division of the process into a separate sample simulation part and sequencing simulation part allows both to develop and evolve independently of each other. Developers can upgrade and develop new library preparation/sequencing simulators to match the current state of sequencing technology, all while still being able to use the same model of input sample. Similarly, as technology improves and we can measure the truth underlying RNA samples with increased fidelity, we can simulate different species of RNA in the input sample, like small RNAs, tRNAs, and circular RNAs, without extensive modification to the sequencing simulation.

The CAMPAREE simulator represents an import first step toward molecule-level simulation. We plan to add further features to improve the simulator and the breadth of biological conditions it can mimic. These include the ability to introduce allele-specific splicing, heterogeneous degradation, editing, and processing of the RNA molecules, use separate parameters to represent nuclear and cytoplasmic RNA fractions, and simulate single-cell samples in addition to bulk RNA. This last option is currently possible as multiple, low expression CAMPAREE runs could each represent the transcriptomes of individual cells before labeling and pooling together. It is our hope that the open source, modular nature of CAMPAREE will allow the community to adapt it to meet their current and future needs. In this way, CAMPAREE can evolve in parallel with our understanding of transcriptional biology.

### Availability and requirements

**Project name:** CAMPAREE.

**Project home page**: https://github.com/itmat/CAMPAREE.

**Operating system:** Tested on GNU/Linux operating systems.

**Programming language:** Python.

**Other requirements:** git, Python 3.6 or higher, Java 1.8.

**License:** GNU General Public License v3.0.

## Supplementary Information

**Additional file 1: Table S1.** Input and output files for each CAMPAREE step. A table listing each step in the CAMPAREE pipeline, along with the input and output files for each. Output files marked with a ‘*’ can be provided by the user to skip the associated step(s).

**Additional file 2: Figure S1.** Flowchart for estimating intronic and intergenic empirical distributions. A flowchart describing how CAMPAREE uses a genome-aligned BAM file and gene annotation to estimate distributions for intron inclusion (sense and antisense) and intergenic expression. This procedure is repeated for each input sample in a CAMPAREE run. Note, a “mintron” is defined as the smallest possible genomic span that does not overlap any exon, intergenic region, or the 1500 bp regions (by default) flanking any transcript’s start and stop coordinates.

**Additional file 3: Figure S2.** Flowchart for estimating transcript, gene, and PSI distributions. A flowchart describing how CAMPAREE estimates distributions for gene-level abundances, transcript-level abundances, and transcript PSI (percent splicing included) values from a FASTA file of transcript sequences and a gene model, both generated from one parental genome, as well as FASTQ files of unaligned input reads. This procedure is repeated for each input sample in a CAMPAREE run.

**Additional file 4: Figure S3.** Flowchart for estimating the distribution of allelic imbalances. A flowchart describing how CAMPAREE estimates the distribution of allelic imbalances (i.e., the percentage of molecules for each gene transcribed from each parental allele). This process uses FASTA files of transcript sequences and gene models generated from both parental genomes, as well as FASTQ files of unaligned reads, and a BAM file containing alignments of reads to the original, source genome. This procedure is repeated for each input sample in a CAMPAREE run.

**Additional file 5: Figure S4.** Empirical distributions estimated by CAMPAREE from real data. (A) Estimated distributions for transcript abundances overlapping intronic regions in the sense orientation (left panel), intronic regions in the antisense orientation (middle panel), and intergenic regions (right panel). (B) Estimated distributions for transcript- and gene-level abundances. Gene-level abundances are calculated by summing the abundances of all transcripts belonging to each gene. (C) Estimated distributions for per-transcript PSI (percent splicing included) values. Data in this figure are for transcripts from genes estimated to express at least one splice form. (D) Estimated distributions for allelic imbalance, represented as the ratio of molecules transcribed from the parent 1 allele and the parent 2 allele. The Y-axes for all plots display the Gaussian kernel density estimates calculated by the density() function in R. The X-axes in panels (A) and (B) display the log10-transformed abundance estimates for each genomic feature in FPK (fragments per kilobase length), a length-normalized measure of transcript abundance. For display purposes, a pseudocount of 0.001 was added to each FPK value, so that features with an FPK of 0 are still displayed in log10 space at the position log10(0 + 0.001) = -3. CAMPAREE estimated these distributions from two real mouse liver RNA-Seq samples (9575 - GSM2599715; 9577 - GSM2599721).

**Additional file 6: Figure S5.** Transcript abundances simulated by BEERS, Polyester, and RSEM after being primed with CAMPAREE output. Scatterplots of transcript abundances simulated by BEERS (left panels), Polyester (middle panels), and RSEM (right panels), compared to CAMPAREE abundances used to prime each RNA-Seq simulator. Data are displayed separately for genes from each parental allele (top and bottom panels). The line of unexpressed transcripts at the bottom of the BEERS panels are from transcripts which BEERS removed from the annotation because they possess genomic features which interfere with BEERS’s underlying simulation (e.g., length < 200 bp, introns < 10 bp). Similarly, the unexpressed transcripts from the bottom of the RSEM panels are from short transcripts. RSEM reads transcript abundances as TPM (transcripts per million) values. The TPM calculation involves calculating a transcript’s “effective length,” by subtracting the estimated RNA-Seq read/fragment length from the transcript’s length. This can result in an effective length < 0 for short transcripts. In the RSEM model, transcripts with effective lengths < 0 have no expression. The X- and Y-axes display the transcript abundances simulated by each RNA-Seq simulator and by CAMPAREE, respectively. For display purposes, a pseudocount of 0.1 was added to each abundance value, so unexpressed transcripts are still displayed in log10 space at the position log10(0 + 0.1) = -1.

**Additional file 7: Figure S6.** Variants introduced by CAMPAREE are maintained in RSEM output. Coverage plots and alignments for reads simulated by RSEM from the two terminal exons of *Polr2j*. Black rectangles highlight variants specific to each parental allele. Red lines on left indicate a ‘T’ substitution present in all alignments from parent 2 allele. Orange lines on the right indicate a ‘G’ substitution present in all alignments from parent 2 allele. Similar results for Polyester and BEERS are displayed in Fig. 4.

## Data Availability

All CAMPAREE data presented in this manuscript were primed with two real RNA-Seq samples, 9576 and 9577, from a previous study [27]. The FASTQ files for these samples are available from the Gene Expression Omnibus (9576 – GSM2599715; 9577 – GSM2599721).

## Abbreviations

RNA-Seq: High-throughput sequencing of RNA
CAMPAREE: Configurable And Modular Program Allowing RNA Expression Emulation
SNV: Single nucleotide variant
PSI: Percent splicing included
iid: Independently and identically distributed
