# Supplementary material for: CAMPAREE: a robust and configurable RNA expression simulator
Source: BMC Genomics. 2021 Sep 25;22:692. doi: 10.1186/s12864-021-07934-2 (PMC8467241; doi:10.1186/s12864-021-07934-2)
Supplement: Supplementary file 1 — Additional file 1: Table S1. Input and output files for each CAMPAREE step. A table listing each step in the CAMPAREE pipeline, along with the input and output files for each. Output files marked with a ‘*’ can be provided by the user to skip the associated step(s). [file 12864_2021_7934_MOESM1_ESM.docx]

Table S1 – Input and output files for each CAMPAREE step

| **Step Name** | **Input Files** | **Output Files** |
| --- | --- | --- |
| GenomeAlignment | - FASTQ files - STAR binary - STAR genome index | - Genome-aligned BAM file* |
| GenomeBamIndex | - Genome-aligned BAM file | - BAM index |
| IntronQuantification | - Genome-aligned BAM file - Reference annotation file | - Intron (sense) distribution - Intron (antisense) distribution - Intergenic distribution |
| VariantsFinder | - Genome-aligned BAM file - Ploidy data - Reference genome sequence | - Subject-specific variants file |
| VariantsCompilation | - All subject-specific variants files - Ploidy data - Reference genome sequence | - Variants VCF file |
| Beagle | - Beagle JAR - Variants VCF file | - Phased variants VCF file* |
| GenomeBuilder | - Ploidy data - Reference genome sequence - Phased variants VCF file | - Parent 1 genome sequence FASTA - Parent 1 indel mapping file - Parent 1 coordinate to reference coordinate mapping - Parent 2 genome sequence FASTA - Parent 2 indel mapping file - Parent 2 coordinate to reference coordinate mapping |
| UpdateAnnotationForGenome | - Reference annotation file - Parent 1/2 indel mapping file - Ploidy data | - Parent 1/2 annotation |
| TranscriptomeFastaPreparation | - Parent 1/2 genome sequence - Parent 1/2 annotation | - Parent 1/2 transcriptome sequence FASTA |
| KallistoIndex | - kallisto binary - Parent 1/2 transcriptome sequence FASTA | - Parent 1/2 kallisto index |
| KallistoQuant | - kallisto binary - Parent 1/2 kallisto index - FASTQ files | - Parent 1/2 kallisto quantifications |
| TranscriptGeneQuantification | - Parent 1 kallisto quantification - Parent 1 annotation | - Transcript distribution - Gene distribution - PSI distribution |
| Bowtie2Index | - Bowtie2 binary - Parent 1/2 transcriptome sequence FASTA | - Parent 1/2 bowtie2 index |
| Bowtie2Align | - Bowtie2 binary - Parent 1/2 bowtie2 index - FASTQ files | - Parent 1/2 transcriptome-aligned SAM |
| AllelicImbalanceQuantification | - Genome-aligned BAM file - Parent 1 annotation - Parent 1 transcriptome-aligned SAM - Parent 2 annotation - Parent 2 transcriptome-aligned SAM | - Allelic imbalance distribution |
| MoleculeMaker | - Gene distribution - Transcript distribution - PSI distribution - Intron (sense) distribution - Allelic imbalance distribution - Parent 1 annotation - Parent 1 genome sequence - Parent 1 indel mapping file - Parent 2 annotation - Parent 2 genome sequence - Parent 2 indel mapping file | - Molecule file |

*Providing this output as an optional file will skip the associated step
