# Supplementary figures and images for: CAMPAREE: a robust and configurable RNA expression simulator
Source: BMC Genomics. 2021 Sep 25;22:692. doi: 10.1186/s12864-021-07934-2 (PMC8467241; doi:10.1186/s12864-021-07934-2)

# Estimating Intronic & Intergenic Distributions

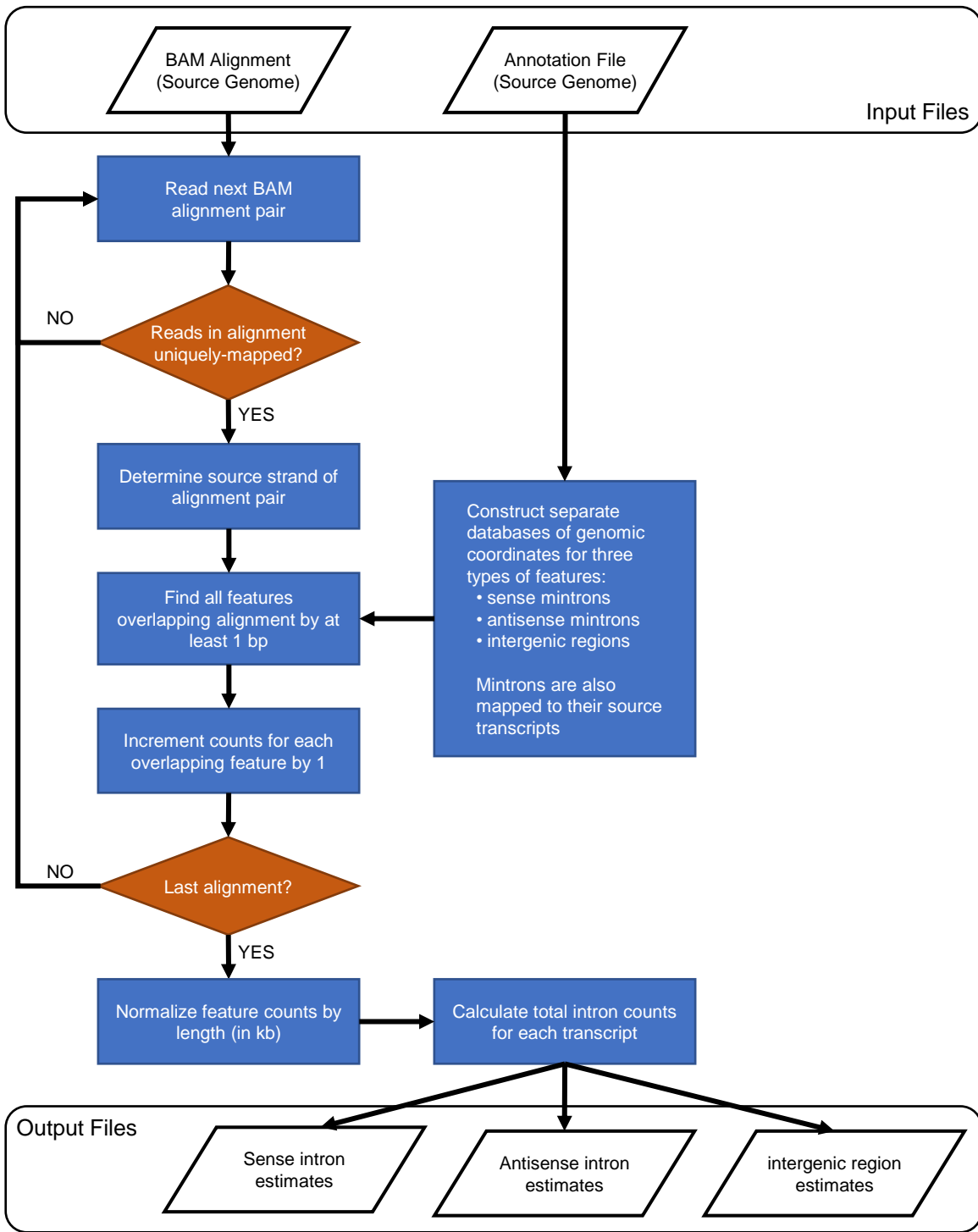

Supplement: Supplementary file 2 — Additional file 2: Figure S1. Flowchart for estimating intronic and intergenic empirical distributions. A flowchart describing how CAMPAREE uses a genome-aligned BAM file and gene annotation to estimate distributions for intron inclusion (sense and antisense) and intergenic expression. This procedure is repeated for each input sample in a CAMPAREE run. Note, a “mintron” is defined as the smallest possible genomic span that does not overlap any exon, intergenic region, or the 1500 bp regions (by default) flanking any transcript’s start and stop coordinates. [file 12864_2021_7934_MOESM2_ESM.pdf]

# Estimating Allelic Imbalance

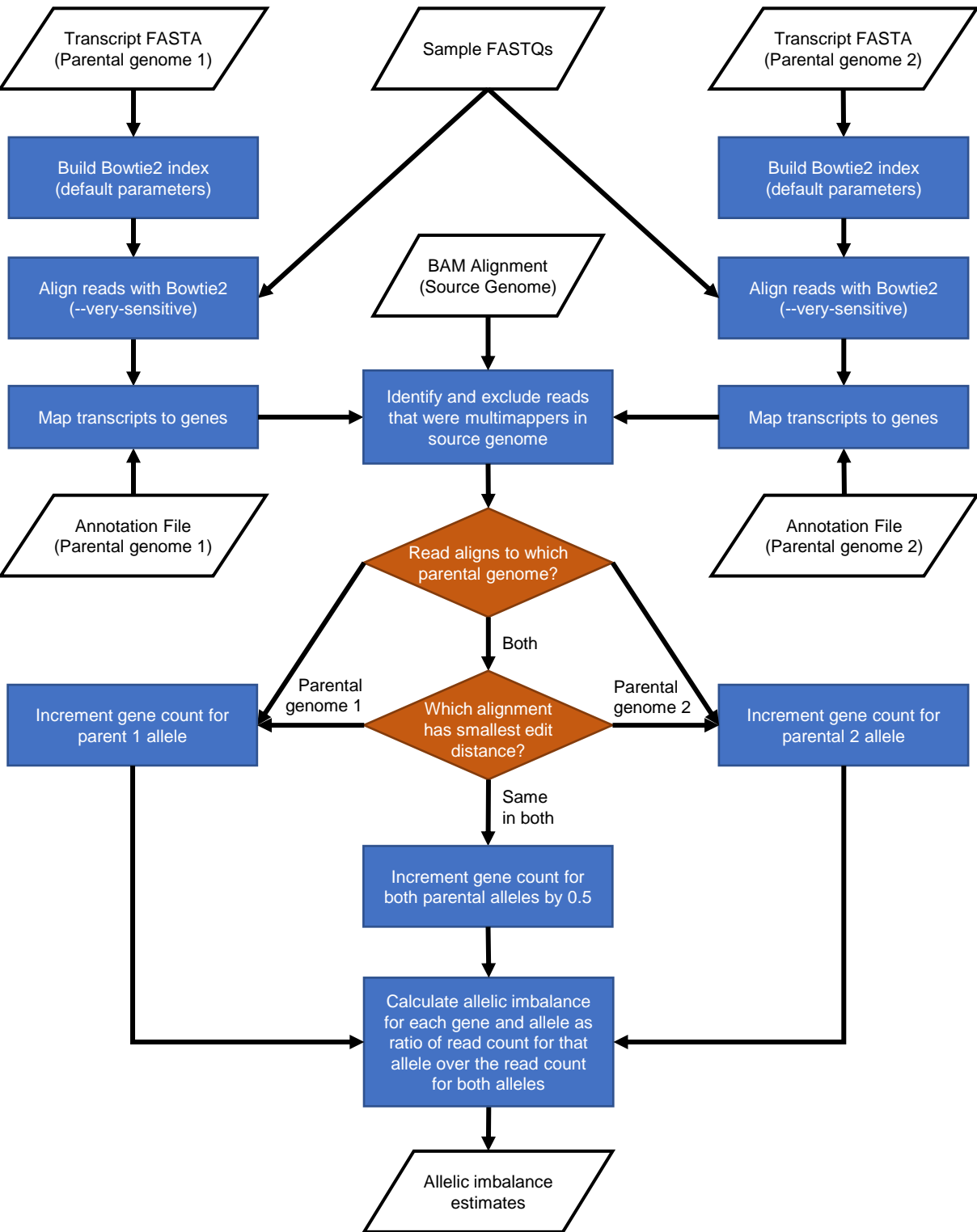

Supplement: Supplementary file 4 — Additional file 4: Figure S3. Flowchart for estimating the distribution of allelic imbalances. A flowchart describing how CAMPAREE estimates the distribution of allelic imbalances (i.e., the percentage of molecules for each gene transcribed from each parental allele). This process uses FASTA files of transcript sequences and gene models generated from both parental genomes, as well as FASTQ files of unaligned reads, and a BAM file containing alignments of reads to the original, source genome. This procedure is repeated for each input sample in a CAMPAREE run. [file 12864_2021_7934_MOESM4_ESM.pdf]

A

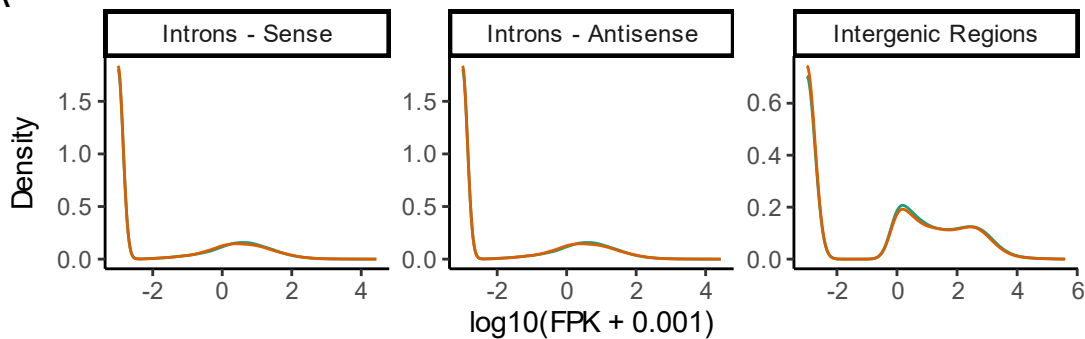

B

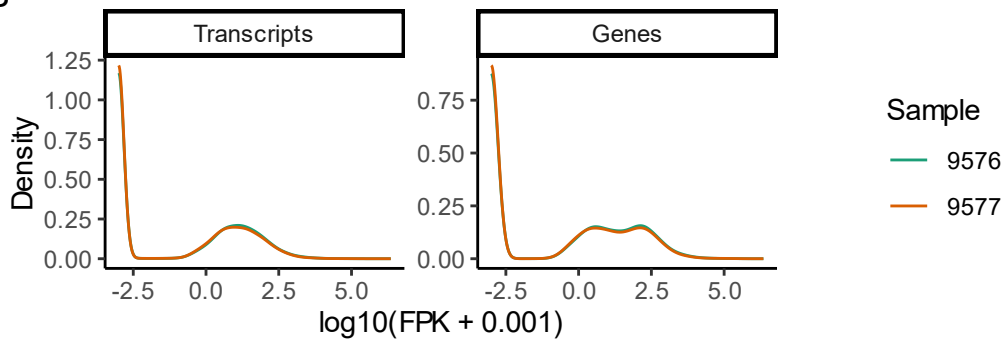

C

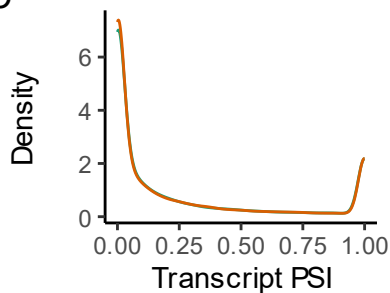

D

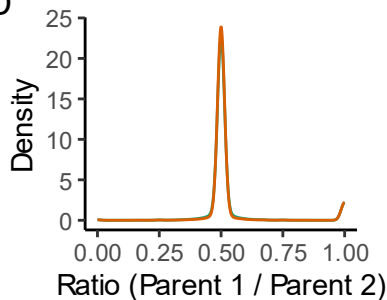

Supplement: Supplementary file 5 — Additional file 5: Figure S4. Empirical distributions estimated by CAMPAREE from real data. (A) Estimated distributions for transcript abundances overlapping intronic regions in the sense orientation (left panel), intronic regions in the antisense orientation (middle panel), and intergenic regions (right panel). (B) Estimated distributions for transcript- and gene-level abundances. Gene-level abundances are calculated by summing the abundances of all transcripts belonging to each gene. (C) Estimated distributions for per-transcript PSI (percent splicing included) values. Data in this figure are for transcripts from genes estimated to express at least one splice form. (D) Estimated distributions for allelic imbalance, represented as the ratio of molecules transcribed from the parent 1 allele and the parent 2 allele. The Y-axes for all plots display the Gaussian kernel density estimates calculated by the density() function in R. The X-axes in panels (A) and (B) display the log10-transformed abundance estimates for each genomic feature in FPK (fragments per kilobase length), a length-normalized measure of transcript abundance. For display purposes, a pseudocount of 0.001 was added to each FPK value, so that features with an FPK of 0 are still displayed in log10 space at the position log10(0 + 0.001) = -3. CAMPAREE estimated these distributions from two real mouse liver RNA-Seq samples (9575 - GSM2599715; 9577 - GSM2599721). [file 12864_2021_7934_MOESM5_ESM.pdf]

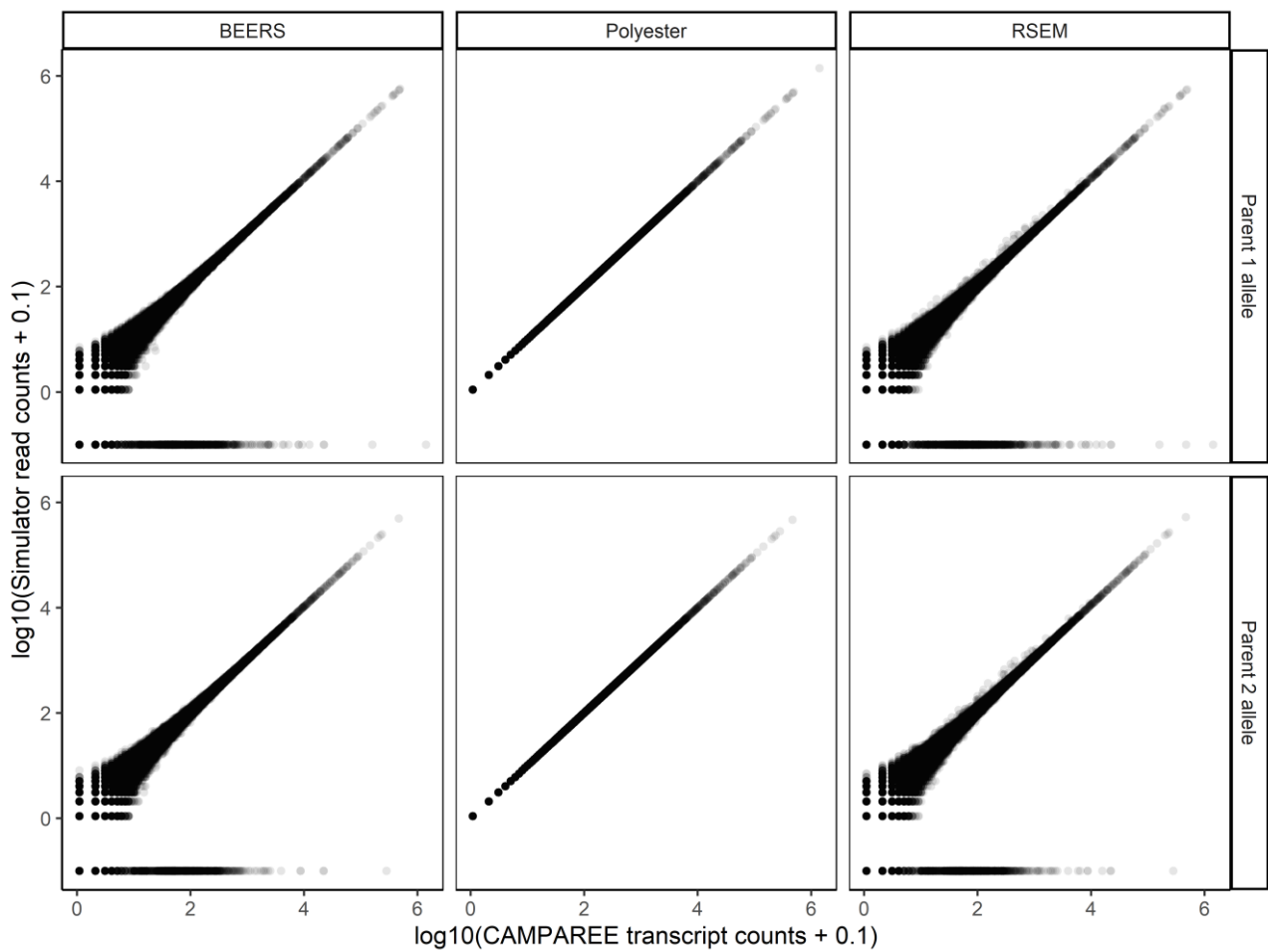

Supplement: Supplementary file 6 — Additional file 6: Figure S5. Transcript abundances simulated by BEERS, Polyester, and RSEM after being primed with CAMPAREE output. Scatterplots of transcript abundances simulated by BEERS (left panels), Polyester (middle panels), and RSEM (right panels), compared to CAMPAREE abundances used to prime each RNA-Seq simulator. Data are displayed separately for genes from each parental allele (top and bottom panels). The line of unexpressed transcripts at the bottom of the BEERS panels are from transcripts which BEERS removed from the annotation because they possess genomic features which interfere with BEERS’s underlying simulation (e.g., length < 200 bp, introns < 10 bp). Similarly, the unexpressed transcripts from the bottom of the RSEM panels are from short transcripts. RSEM reads transcript abundances as TPM (transcripts per million) values. The TPM calculation involves calculating a transcript’s “effective length,” by subtracting the estimated RNA-Seq read/fragment length from the transcript’s length. This can result in an effective length < 0 for short transcripts. In the RSEM model, transcripts with effective lengths < 0 have no expression. The X- and Y-axes display the transcript abundances simulated by each RNA-Seq simulator and by CAMPAREE, respectively. For display purposes, a pseudocount of 0.1 was added to each abundance value, so unexpressed transcripts are still displayed in log10 space at the position log10(0 + 0.1) = -1. [file 12864_2021_7934_MOESM6_ESM.pdf]

RSEM

$p = 2.03 \times 10^{-6}$

Parent 1 allele

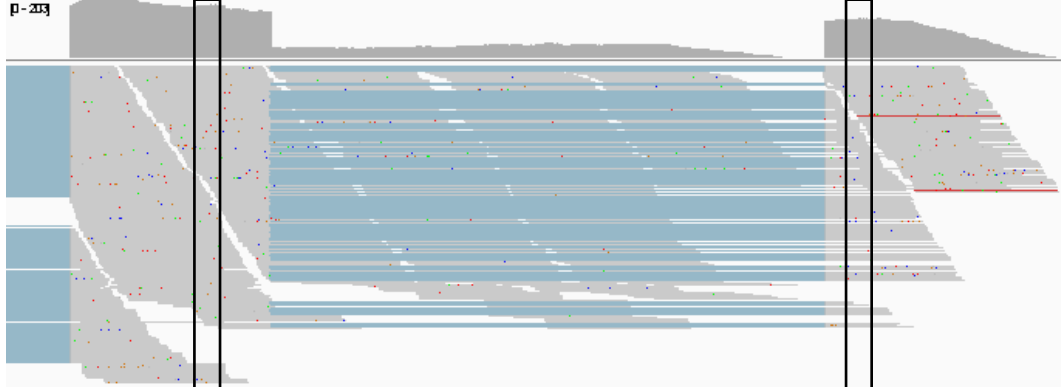

$p = 6.3 \times 10^{-6}$

Parent 2 allele

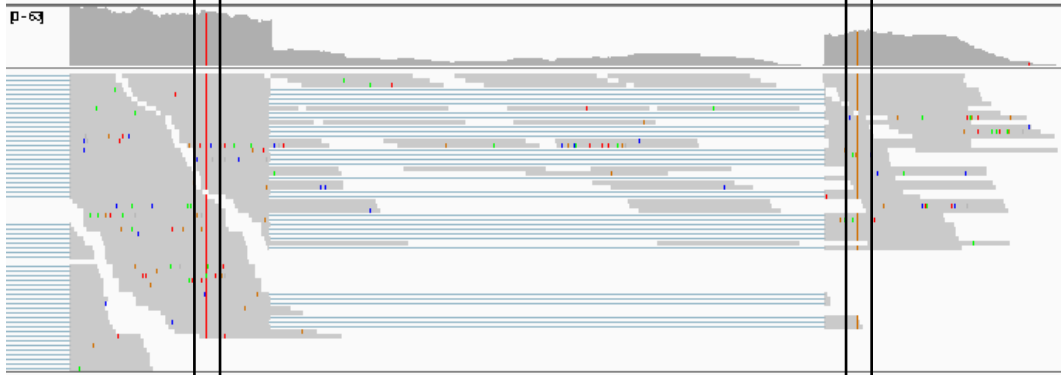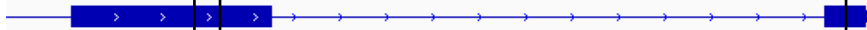

Supplement: Supplementary file 7 — Additional file 7: Figure S6. Variants introduced by CAMPAREE are maintained in RSEM output. Coverage plots and alignments for reads simulated by RSEM from the two terminal exons of Polr2j. Black rectangles highlight variants specific to each parental allele. Red lines on left indicate a ‘T’ substitution present in all alignments from parent 2 allele. Orange lines on the right indicate a ‘G’ substitution present in all alignments from parent 2 allele. Similar results for Polyester and BEERS are displayed in Fig. 4. [file 12864_2021_7934_MOESM7_ESM.pdf]
