# Supplementary material for: CAMPAREE: a robust and configurable RNA expression simulator
Source: BMC Genomics. 2021 Sep 25;22:692. doi: 10.1186/s12864-021-07934-2 (PMC8467241; doi:10.1186/s12864-021-07934-2)
Supplement: Supplementary file 3 — Additional file 3: Figure S2. Flowchart for estimating transcript, gene, and PSI distributions. A flowchart describing how CAMPAREE estimates distributions for gene-level abundances, transcript-level abundances, and transcript PSI (percent splicing included) values from a FASTA file of transcript sequences and a gene model, both generated from one parental genome, as well as FASTQ files of unaligned input reads. This procedure is repeated for each input sample in a CAMPAREE run. [file 12864_2021_7934_MOESM3_ESM.pdf]

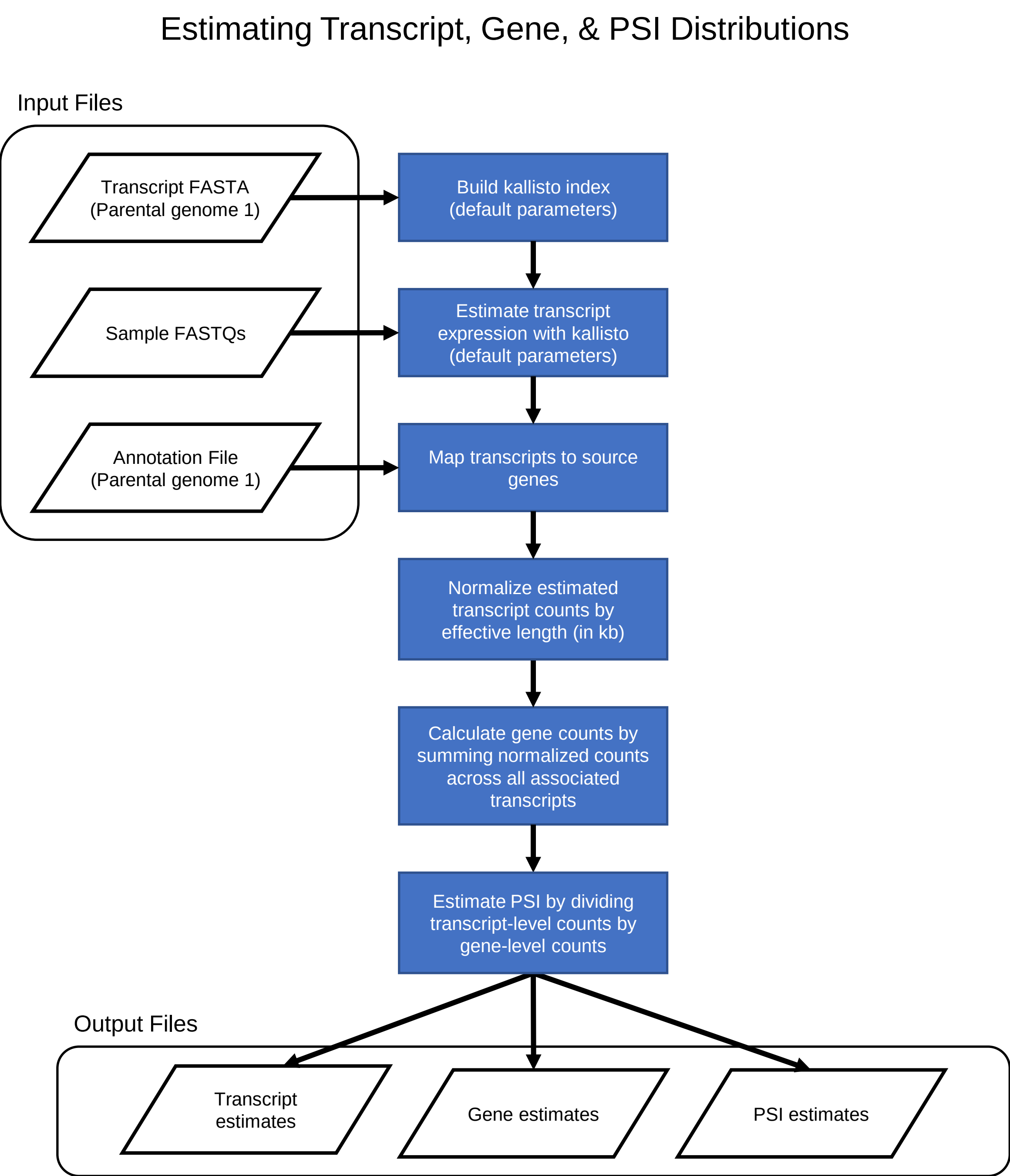
Estimating Transcript, Gene, & PSI Distributions
Input Files
Transcript FASTA (Parental genome 1)
Sample FASTQs
Annotation File (Parental genome 1)
Build kallisto index (default parameters)
Estimate transcript expression with kallisto (default parameters)
Map transcripts to source genes
Normalize estimated transcript counts by effective length (in kb)
Calculate gene counts by summing normalized counts across all associated transcripts
Estimate PSI by dividing transcript-level counts by gene-level counts
Output Files
Transcript estimates
Gene estimates
PSI estimates
